# Template Free and Binderless NiO Nanowire Foam for Li-ion Battery Anodes with Long Cycle Life and Ultrahigh Rate Capability

**DOI:** 10.1038/srep29183

**Published:** 2016-07-18

**Authors:** Chueh Liu, Changling Li, Kazi Ahmed, Zafer Mutlu, Cengiz S. Ozkan, Mihrimah Ozkan

**Affiliations:** 1 Materials Science and Engineering Program, University of California, Riverside, CA, USA; 2 Department of Electrical Engineering, University of California, Riverside, CA, USA; 3 Department of Mechanical Engineering, University of California, Riverside, CA, USA

## Abstract

Herein, NiO-decorated Ni nanowires with diameters ca. 30–150 nm derived from Ni wire backbone (ca. 2 μm in diameter) is directly synthesized on commercially available Ni foam as a renovated anode for Li-ion batteries. Excellent stability with capacity 680 mAh g^−1^ at 0.5C (1C = 718 mA g^−1^) is achieved after 1000 cycles. Superior rate capability is exhibited by cycling at extremely high current rates, such as 20C and 50C with capacities ca. 164 and 75 mAh g^−1^, respectively. The capacity can be recovered back to ca. 430 mAh g^−1^ in 2 cycles when lowered to 0.2C and stably cycled for 430 times with capacity 460 mAh g^−1^. The NiO nanowire foam anode possesses low equivalent series resistance ca. 3.5 Ω, resulting in superior power performance and low resistive losses. The NiO nanowire foam can be manufactured with bio-friendly chemicals and low temperature processes without any templates, binders and conductive additives, which possesses the potential transferring from lab scale to industrial production.

In recent times, electric vehicles (EVs)^1^ are vigorously investigated and developed to diminish the dependence on fossil fuels and alleviate the deterioration of natural environment. Hybrid (HEV) and plug-in (PEV) hybrid EVs^2^ utilizing both batteries and internal combustion engines (ICEs) can partially resolve these issues, but the consumption of gasoline and emission of greenhouse gases from ICEs still remain problematic. Pure EVs powered by purely lithium ion batteries (LIBs) can totally eliminate these difficulties. However, the cruise range of pure EVs is still limited, such as ca. 300 miles per charge of Tesla Model S^3^. Accordingly, it is crucial to improve the capacity and energy density of LIBs while maintaining the power density simultaneously. Capacity of traditional graphite anode with potential ca. 0.2 V vs. Li is limited to theoretically 372 mAh g^−1^ and practically ca. 310 mAh g^−1^ for LiC_6_ as a result of intercalation reactions^2^. Higher energy density and capacity can be reached by utilizing conversion reactions of metal oxides, such as FeO, CoO, NiO and CuO^4^,^5^, in potential range 0.01–3 V vs. Li with ca. 700 mAh g^−1^ by the equation MO + 2Li^+^ + 2e^−^ = M + Li_2_O^2^,^6^. Among these, NiO is appealing owing to its high theoretical capacity (718 mAh g^−1^), environmental benignity and low cost^6^. Nonetheless, it still suffers from low cycling stability and low rate capability resulting from large volume expansion and poor electrical conductivity, respectively^7^.

To overcome these barriers, various NiO nanostructures have been developed to accommodate mechanical strain during cycling, to improve electrical contact and shorten ion diffusion length to reduce resistivity^6^,^7^,^8^,^9^. Three-dimensional curved NiO nanomembranes synthesized by electron beam evaporation demonstrate high capacity (721 mAh g^−1^) at 1.5C over 1400 cycles and high rate capability at 50C with ca. 60 mAh per gram^8^. However, costly processes relying on high vacuum system prevent it from large scale production. NiO nanorods anchored on Ni foam by anodization in oxalic acid at 50 V followed by annealing in air at 400 °C exhibit 706 mAh g^−1^ at 1A per gram^7^. Nevertheless, high voltage anodization utilizing electricity renders the process expensive. Relatively thick wall of the nanorods (200–500 nm) result in rapid Coulombic efficiency drop to ca. 98% after only 70 cycles^7^. NiO nanofibers with diameters ca. 100 nm prepared by electrospinning and air annealing at 800 °C show maximum capacity 784 mAh g^−1^ at 80 mA g^−1^ with low capacity retention (ca. 75%) after 100 cycles^6^. The addition of carbon additive and binder further decrease the specific capacity of the electrode. Ni/NiO nanofoam with skeleton diameter 200–300 nm formed by burning nickel nitrate with 2-methoxyethanol followed by oxidation at 350 °C leads to 835 mAh g^−1^ at 0.5C after 200 cycles^9^. However, long cycle stability is still questionable since capacity retention is only 85% after 200 cycles. Accordingly, NiO nanostructures with high capacity, energy density, rate capability and cycling stability are still highly desired.

Since NiO can be derived from Ni metal simply by annealing in air, NiO nanostructures can be obtained if Ni can be fabricated into nano-sized framework^9^,^10^. Commercial nano-Ni foam composed of Ni nanowires deposited with SnO_2_ by atomic layer deposition is shown to produce good stability, high capacity and rate performance in Li-ion anode^11^. Nanofoams composed of Ni nanowires (100–1000 nm dia.) can be created by refluxing glycerol and nickel acetate (Ni(Ac)_2_) at ca. 300–400 °C and atmospheric pressure^10^,^12^. Surface area of Ni metal can be further enlarged to Ni oxalate nanowires or nanosheets by oxalic acid etching^13^, while Ni metal phase can be resumed by annealing Ni oxalate under reducing or inert atmospheres^14^,^15^,^16^. In this work, NiO-decorated Ni nanowires (dia. ca. 30–150 nm) derived from micro-sized Ni wire backbone (ca. 2 μm in dia.) is directly grown on Ni foam as an innovated anode for Li-ion batteries. Micro-sized Ni wires are synthesized on Ni foam by heating with Ni(Ac)_2_/glycerol solution at 400 °C. Ni oxalate nanoneedles (dia. ca. 30–70 nm) are derived from Ni wires by oxalic acid treatment at 80 °C to further increase the surface area of the electrode. Ni nanowires can be obtained by reducing Ni oxalate nanoneedles with hydrogen at 350 °C. NiO is formed on Ni nanowires by calcination in air from 350 to 450 °C. This NiO anode demonstrates high stability with capacity 680 mAh g^−1^ after 1000^th^ cycle at 0.5C, where 1C = 718 mA g^−1^. Even though the anode is cycled at extremely high current rate, such as 20C and 50C, the capacities can still be ca. 164 and 75 mAh g^−1^, respectively, which exhibit the good rate capability of this nanostructured NiO anode. This carbon-less and binder-less NiO nanowire foam (NWF) anode possesses low equivalent series resistance (ESR) ca. 3.5 Ω, resulting in superior power performance and low resistive losses. The NiO NWF can be manufactured with eco-friendly chemicals, low temperature processes without any templates, binders and conductive additives, which might be easily transferred from lab scale to massive production.

## Methods

### Materials synthesis

Ni foam (MTI Corp., EQ-bcnf-16 m) with 15 mm diameter was first flattened to thickness ca. 120 μm. Flat Ni foam was immersed in a 10 ml beaker filled with a solution of 2.5 ml 0.08 M nickel acetate tetrahydrate (Ni(Ac)_2_•4 H_2_O, Sigma-Aldrich, 98%) in glycerol (Acros, 99+%) heated at 400 °C on a hotplate for 40 min without stirring with Al foil cover to prevent excess solvent evaporation^10^. After growth, Ni wires attached on Ni foam were washed with deionized water 15 times to remove extra glycerol and Ni ions, and dried on a hotplate at 120 °C for 5 min. Magnetic stir rotor inside the hotplate provided the magnetic field for the alignment of Ni wire growth. Total Ni wire weight attached on Ni foam was ca. 7.5 to 8 mg. Freshly prepared 2 ml solution of 0.3 M oxalic acid dihydrate (ICN Biomedicals Inc., reagent grade) in ethanol (Decon Lab. Inc., 200 proof) with 10 wt% deionized water was used to etch the Ni wires at 80 °C for 1.5 h on hotplate into Ni oxalate needle-like nanostructures^13^, which were further reduced back to Ni nanowires in a tube furnace with H_2_ (50 sccm), Ar (100 sccm) at 20 torr for 10 min at 350 °C with ramping rate 30 °C min^−1^. For NiO growth, Ni nanowires attached on Ni foam were heated in a tube furnace flowed with air from room temperature to 450 °C with ramping rate 2 °C min^−1^, and the NiO-decorated Ni NWF electrode was taken out of the furnace immediately without holding at elevated temperature to control the oxide loading. NiO loading was equal to Δm ^*^ [M.W. of NiO]/[M.W. of O] = Δm * 74.69/16, where Δm is the weight difference of the electrode before and after oxidation according to the reaction 2Ni + O_2_ = 2NiO^9^. NiO loading was ca. 0.5 mg cm^−2^ per electrode.

### Materials characterization

Surface morphology and elemental analysis were performed by scanning electron microscopy (SEM, FEI NovaNanoSEM 450) with energy dispersive X-ray spectroscopic (EDX) detector. Crystal structures were examined by X-ray diffraction (XRD, PANalytical Empyrean) with Cu-Kα radiation. Raman spectroscopy (Renishaw DXR) utilizing 532 nm laser with 8 mW excitation power and 100x objective lens was used to characterize NiO NWF electrode. Chemical states of NiO were examined by X-ray photoelectron spectroscopy (XPS, Kratos AXIS ULTRA DLD XPS system) with Al Kα monochromated X-ray source and 165-mm mean radius electron energy hemispherical analyzer. Thermogravimetric analysis (TGA, TA instruments, SDT Q600) was performed on the electrode with air from room temperature to 700 °C with 2 °C min^−1^ to determine the weight change and oxidation temperature of the NiO NWF electrode. N_2_ adsorption/desorption for Brunauer-Emmett-Teller (BET) surface were measured on NiO NWF electrodes at 77 K on a Micromeritics ASAP 2020 analyzer.

### Electrochemical Impedance Spectroscopy

Electrochemical performance of the NiO NWF anode was evaluated in two-electrode half-cell configuration with Li foil (MTI Corp.) counter electrode in CR2032 coin cell (MTI Corp.) using electrolyte comprising 1 M LiPF_6_ (Sigma-Aldrich, battery grade) in fluoroethylene carbonate (FEC, Sigma-Aldrich, 99%) and dimethyl carbonate (DMC, Sigma-Aldrich, anhydrous) in FEC:DMC 1:1 (v/v) ratio. Cells were assembled in an Ar filled glovebox (VAC Omni-lab) with moisture and oxygen concentration below 1 ppm. Porous membrane (Celgard 3501) was used as the separator. Cyclic voltammetry (CV) was scanned at 0.1 mV s^−1^ in the range 3.0 to 0.02 V (vs. Li/Li^+^) with Biologic VMP3. Galvanostatic charge-discharge and cycling measurements were investigated in 3.0 to 0.02 V (vs. Li/Li^+^) with various current rates by Arbin BT2000. Electrochemical impedance spectroscopy (EIS) at E_we_ = 3.0 V (vs. Li/Li^+^) between 10 mHz to 1 MHz with amplitude 10 mV were performed with Biologic VMP3.

## Results and Discussion

**Figure 1:**
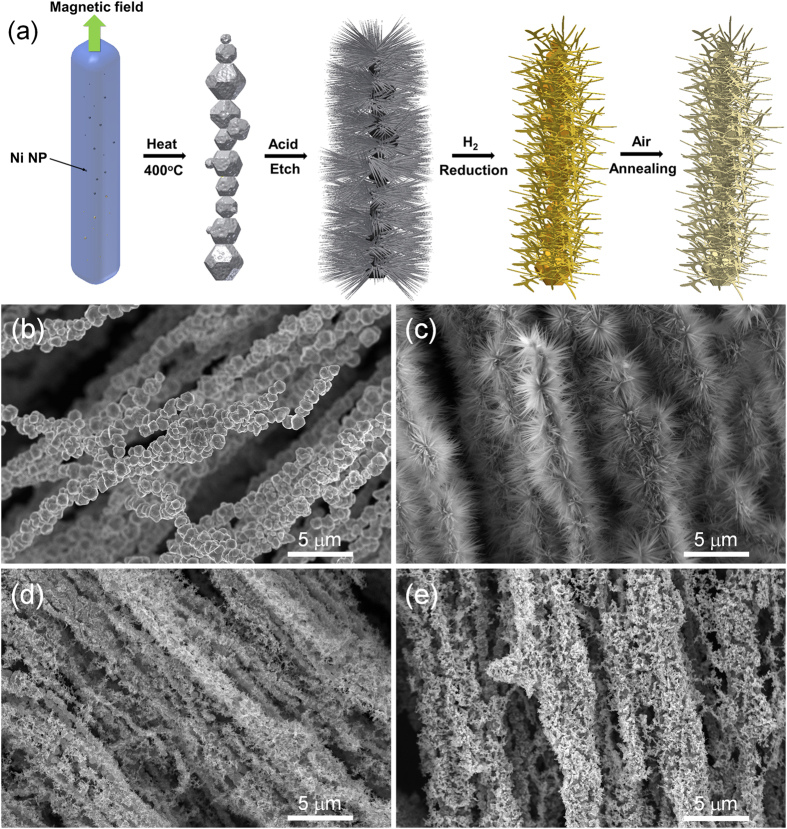
(**a**) Schematic of synthesis of NiO nanowire foam (NWF). SEM images of (**b**) Ni wires, (**c**) Ni oxalate nanoneedles, (**d**) Ni nanowires and (**e**) NiO NWF.

**Figure 2:**
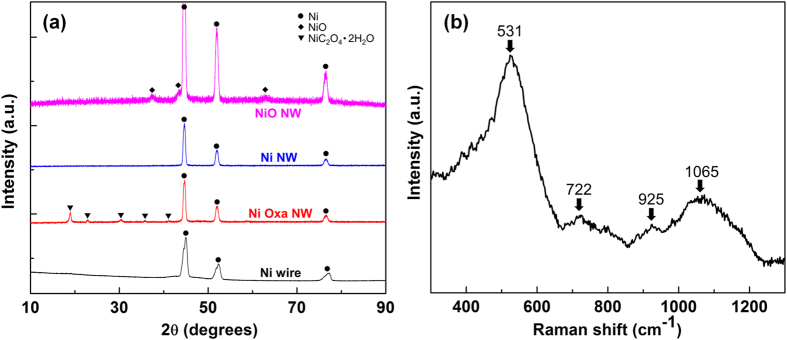
(**a**) XRD patterns of Ni wire, Ni oxalate nanowire (Ni Oxa NW), Ni nanowire (Ni NW) foam, and NiO NW foam. (**b**) Raman spectrum of the NiO NWF.

Porous NiO NWF anode with large surface area can accommodate volume expansion during lithiation/delithiation, allow fast lithium ion transportation and provide intimate contact between the active materials and the current collector. Synthetic procedures and the scanning electron microscopic (SEM) images of the NiO NWF are shown in Fig. 1. Ni foam is directly immersed in a solution of 0.08 M Ni(Ac)_2_/glycerol at 400 °C and atmospheric pressure. Ni^2+^ ions reduced by glycerol nucleate into Ni polyhedral nanoparticles which are further grown into micro-sized Ni wires under the magnetic field of the magnetic stir rotor inside the hotplate (Fig. 1a,b). The practicability of template-less and self-assembled synthesis of ferromagnetic materials into nanowires has been shown by metallic Ni nanowire nonwoven clothes as potential NiO anode for Li-ion batteries^17^. The as-synthesized Ni wires demonstrate X-ray diffraction (XRD) peaks at 45.1°, 52.5° and 76.9°, revealing the characteristic of Ni metal phase (ref. code: 01-070-0989, Fig. 2a). The surface area of the Ni wires can be increased by etching in 0.3 M oxalic acid/ethanol solution with 10 wt% water at 80 °C (Fig. 1c) by the formation of Ni oxalate nanowires, the existence of which is indicated by XRD peaks at 18.9°, 23.0°, 30.4°, 35.8°, and 41.2° (Fig. 2a, NiC_2_O_4_•2 H_2_O, ref. code 00-014-0742). By hydrogen reduction at 350 °C, Ni oxalate can transform completely into Ni nanowire (Fig. 1d, Supplementary Fig. S1a,c) with the XRD patterns showing only Ni metallic phase without the presence of Ni oxalate (Fig. 2a). By annealing in air to 450 °C, the Ni NWF can be covered with NiO layer (Fig. 1e, Supplementary Fig. S1b,d), XRD patterns of which show peaks at 37.2°, 43.4° and 63.0° (Fig. 2a, ref. code 01-071-6719) along with the metallic Ni patterns from the underlying Ni nanowires and Ni foam. Nitrogen adsorption–desorption isotherms and pore size distribution of NiO NWF (Supplementary Fig. S1e,f) exhibit mesoporous nature of the active materials. BET surface area of the NiO NWF is 143.43 m^2^ g^−1^, demonstrating the high specific surface area of the electrode. Uniform distribution of NiO on the Ni NWF is displayed by energy dispersive X-ray spectroscopic (EDX) analysis and elemental mapping (Supplementary Fig. S2). The existence of NiO is further demonstrated by Raman spectrum (Fig. 2b) showing four broad peaks corresponding to one-phonon longitudinal optical mode (LO at 531 cm^−1^)^8^, two-phonon transverse optical mode (2TO at 722 cm^−1^)^18^, TO + LO (at 925 cm^−1^)^18^ and 2LO (at 1065 cm^−1^)^8^ modes. Thermogravimetric analysis (TGA, Supplementary Fig. S3) indicates NiO formation starts from ca. 350 °C and the Ni NWF is continuously oxidized with elevated temperature.

**Figure 3:**
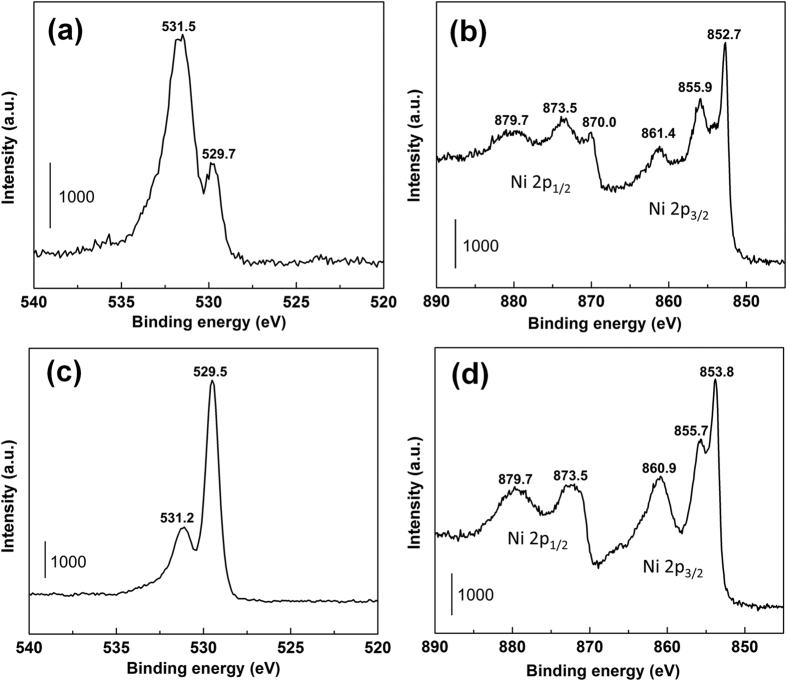
XPS spectrum of Ni NWF for (**a**) O 1s and (**b**) Ni 2p levels, and of NiO NWF for (**c**) O 1s and (**d**) Ni 2p levels.

Ni and NiO NWF are investigated by X-ray photoelectron spectroscopy (XPS) to determine the valence states and composition with O 1s and Ni 2p core levels (Fig. 3). For Ni NWF, O 1s peaks (Fig. 3a) at ca. 531.5 eV and 529.7 eV are attributed to Ni^3+^ from Ni_2_O_3_ and Ni^2+^ from NiO, respectively, with stronger intensity from Ni^3+^ since a Ni_2_O_3_ layer tends formed on metallic nickel in air^19^. For NiO NWF, the O 1s peak of Ni^2+^ at ca. 529.5 eV shows stronger signals than that of Ni^3+^ at ca. 531.2 eV (Fig. 3c), demonstrating that NiO is the dominant species after air annealing. Signals from 870–885 eV and 850–865 eV correspond to Ni 2p_1/2_ and Ni 2p_3/2_ levels, respectively^8^. Metallic Ni peak detected in Ni NWF at ca. 852.7 eV^19^ is absent in NiO NWF, indicating good oxide layer coverage after oxidation without metallic backbone exposed. Peaks at 855.9 eV and 861.4 eV in Ni 2p_3/2_ for Ni NWF further demonstrate Ni_2_O_3_ is the main surface composition, while peaks at 853.8, 855.7 and 860.9 eV for NiO NWF can be mainly attributed to Ni^2+^ from NiO^19^. Survey spectrum of Ni and NiO NWF are shown in Supplementary Fig. S4.

**Figure 4:**
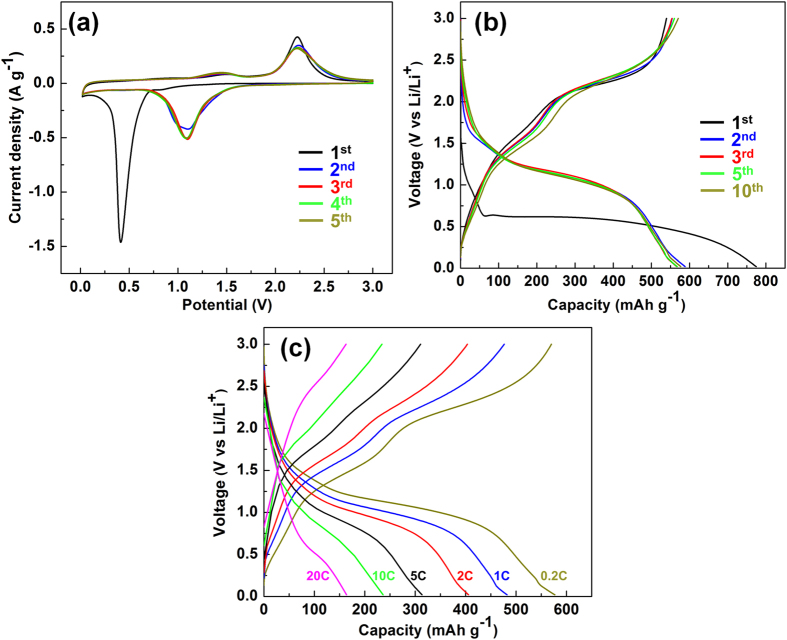
(**a**) Cyclic voltammetric diagrams of the NiO NWF anode for 5 cycles with 0.1 mV s^−1^. Charge-discharge curves of (**b**) selected cycles at 0.2C (1 C = 718 mA g^−1^) and (**c**) at various C rates.

NiO NWF is tested as an anode in a two-electrode half-cell configuration with Li foil as the counter electrode. Cyclic voltammetric (CV) profiles are measured in the potential window 0.02–3.0 V at the scan rate 0.1 mV s^−1^ (Fig. 4a). In the cathodic scan of the first cycle, an intense peak at ca. 0.41 V is attributed to the formation of solid electrolyte interface (SEI) layer, initial reduction of NiO and formation of Li_2_O (NiO + 2Li^+^ + 2e^−^ → Ni + Li_2_O)^8^,^9^,^20^. In the following cycles, this discharge peak potential becomes weaker and move to ca. 1.09 V^8^,^9^. During anodic scan, the broad peak at ca. 1.45 V and the stronger peak at ca. 2.23 V correspond to SEI layer decomposition and NiO formation/Li_2_O decomposition (Ni + Li_2_O → NiO + 2Li^+^ + 2e^−^), respectively^8^,^9^,^20^. Charge-discharge potential curves of the 1^st^ to 10^th^ cycles of the NiO NWF anode within the 0.02–3.0 V voltage window at the current rate 0.2C (1C = 718 mA g^−1^) are shown in Fig. 4b. For the 1^st^ discharge, a potential plateau at ca. 0.6 V is observed. For the subsequent cycles, the discharge potentials is shifted to a slope from ca. 1.6 to 1.0 V, while the charge potential plateaus at ca. 1.5 V and 2.2 V are maintained, which is consistent with the CV results. The 1^st^ cycle Coulombic efficiency (CE) of 70.0% calculated from 1^st^ cycle charge capacity (541 mAh g^−1^) divided by 1^st^ cycle discharge capacity (773 mAh g^−1^) can be attributed to the formation of SEI layer between the active materials and the electrolyte^8^,^9^,^10^. The NiO NWF anode demonstrates discharge (577 mAh g^−1^) and charge capacity (570 mAh g^−1^) of the 10^th^ cycle for CE of 98.8%, suggesting good recyclability of the electrode.

**Figure 5:**
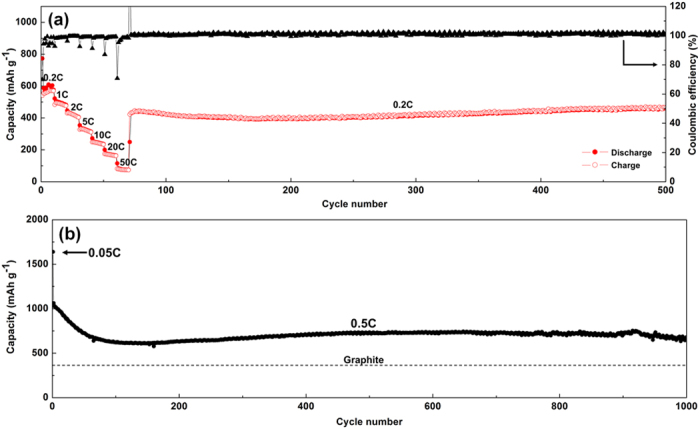
(**a**) Rate capability of the NiO NWF anode with C rates ranging from 0.2C to 50C, and stability at 0.2C for 430 cycles. (**b**) Discharge capacity of the NiO NWF anode started with 0.05C followed by 0.5C for 1000 cycles comparing with the capacity of graphite.

Charge-discharge voltage curves at various current rate from 0.2C to 20C are shown in Fig. 4c. Higher overpotentials (lower discharge and higher charge potentials) observed for higher C rates can be attributed to the kinetic effect of the electrode^8^. Similar curve shapes regardless of current density suggest good stability under high C rates. Discharge capacities at 0.2C, 1C, 2 C, 5C, 10C and 20C are 577, 482, 406, 313, 236 and 164 mAh g^−1^, respectively (Fig. 5a). Impressively, even at extremely high current rate (50C = 35900 mA g^−1^), the capacity can still reach 75 mAh g^−1^. The capacity can be resumed back to ca. 430 mAh g^−1^ in 2 cycles when the current rate is lowered to 0.2C, and the NiO NWF anode can still be stable for 430 cycles with 460 mAh g^−1^ at 500^th^ cycle with Coulombic efficiency fluctuates between 99.5–102.6%. The capacity recovery with lower current density can be explained as lower overpotentials of the charge-discharge process leading to longer charge-discharge time and thus larger capacities in a fixed potential range (i.e. 0.02–3.0 V), which is also observed in the literatures^8^,^21^,^22^,^23^,^24^. Failure of the capacity recovery to the values of 0.2C in the first 10 cycles might be attributed to the nanostructural change after cycling at ultra large current densities (e.g. 20C and 50C). However, the porous nature of the nanowire architecture can accommodate the mechanical strain to a certain degree and maintain its capacities in the following cycling process. The gradual increase in capacity and CE higher than 100% can be attributed to the activation of anode materials after cycling^8^,^25^. The superior rate capability can be ascribed to the intimate electrical contact between NiO active materials and the conductive metallic Ni support, and the porous framework providing access for the electrolyte resulting in short ionic diffusion length^8^. Compared to the 482 mAh g^−1^ at 0.72 A g^−1^ of NiO NWF, recent studies^26^,^27^ demonstrate higher capacities at similar current densities by decorating NiO nanosheets onto porous carbon supports, such as sulfonated polystyrene hollow particles (SPS)^26^ with 736 mAh g^−1^ at 0.8 A g^−1^ and carbon rods (CMK-3)^27^ with 824 mAh g^−1^ at 0.8 A g^−1^. Even though the high specific surface area of carbon supports promotes the rate capabilities of NiO, relatively lower 1^st^ cycle Coulombic efficiencies (67% of SPS, ~30–50% of CMK-3) suggest larger amount of SEI layer formation during the first cycle leading to irreversible Li consumption of the cathode materials (e.g. LiCoO_2_) and permanent capacity loss when utilized in full cells. Accordingly, improving rate capabilities without compromising 1^st^ cycle Coulombic efficiencies still remains challenging of the state-of-the-art NiO electrodes.

Cycling stability of the NiO NWF anode is further examined by cycling at 0.05C for the first cycle followed by 0.5C for 999 cycles in the potential window 0.02–3.0 V (Fig. 5b). Superior cycling performance is shown by initially capacity fading to 620 mAh g^−1^ in 100 cycles, gradually increasing to 735 mAh g^−1^ at the 500^th^ cycle, holding steadily to 717 mAh g^−1^ at the 900^th^ cycle, and then slowly decreasing to 680 mAh g^−1^ at the 1000^th^ cycle, which is notably close to the theoretical value of NiO (718 mAh g^−1^) and larger than traditional graphite^28^ (372 mAh g^−1^) anode. Representative charge-discharge curves from the 100^th^ to 500^th^ cycles (Supplementary Fig. S5a) indicate gradual material activation with less polarization of delithiation. After 500 cycles, discharge curves start to lose the plateau with more polarization^8^, suggesting higher energy necessary for lithiation, while similar charge curves are maintained until reaching the 1000^th^ cycle (Supplementary Fig. S5b).

Higher NiO loading (1.3 mg cm^−2^) can be achieved with ramping rate 30 °C min^−1^ to higher temperature (500 °C) and kept at 500 °C for 1 h in air. Compared to 0.5 mg cm^−2^, high temperature and elongated annealing time lead to higher NiO loading with stronger NiO reflections (Supplementary Fig. S6a). Discharge capacities of 1.3 mg cm^−2^ NiO (Supplementary Fig. S6b) are similar to 0.5 mg cm^−2^ at 1C and 2C. Nonetheless, capacities diminish to lower values after 5C, which is attributed to the resistive nature of thicker NiO layer resulting in higher overpotentials at large current densities. Higher loading compromises the rate capability of the electrode. Accordingly, to further increase the areal loading, structural optimization could be performed on the NiO NWF, such as increasing areal density of Ni wire backbone by surface treatment of Ni foam^16^, decreasing the diameter of Ni wire by variation of synthetic temperature or Ni(Ac)_2_ concentrations^10^,^12^, and water concentrations during oxalic acid etching^13^ to modify Ni oxalate nanostructures in the future study.

Superior stability of the NiO NWF is demonstrated in the SEM images after 1000 cycles (Supplementary Fig. S6c–e). Main structure consisting of Ni wire backbone is still intact (Supplementary Fig. S6c–d). While the void space between NiO nanowires is filled with SEI products (Supplementary Fig. S6e), the shape of nanowires is discernible without obvious cracking or detachment, which further elucidate the ability of accommodation of mechanical strain of the NiO NWF electrode.

**Figure 6:**
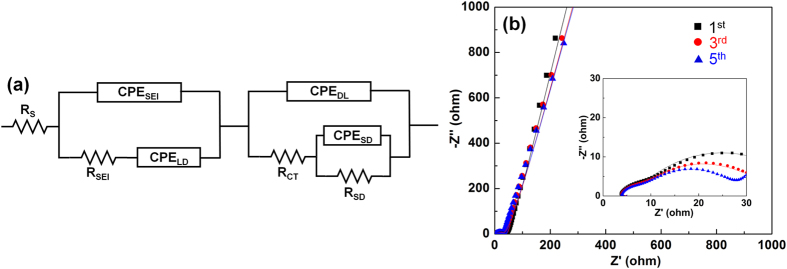
EIS analysis of the NiO NWF with (**a**) the equivalent circuit and (**b**) the Nyquist plots of experimental results (solid symbols) and fitted results (solid lines) after 1, 3 and 5 CV cycles.

Electrochemical impedance spectroscopy (EIS) are performed to further verify the superior electrochemical performance of the NiO NWF anode after one, three and five CV cycles (Fig. 6). The equivalent circuit shown in Fig. 6a is used to fit the impedance data. Experimental results shown in solid symbols (Fig. 6b) are fitted by straight lines using parameters shown in Table 1. Constant phase elements (CPEs) describing non-ideal capacitances with parameters Q analogous to capacitance and the ideality factor n are necessitated due to the existence of spatial and chemical non-uniformity across the electrode and the solid electrolyte interphase (SEI) surface^29^,^30^. R_S_ is the equivalent series resistance (ESR)^31^,^32^,^33^, which represents resistances of electrolyte, metallic leads^34^, cell hardware, current collectors and electrode materials^35^. The first parallel impedance branch in the equivalent circuit describes the SEI layers (R_SEI_ + CPE_SEI_) and diffusion of lithium ions in liquid phase near the electrode surface (CPE_LD_). The second impedance branch accounts for double-layer impedance (CPE_DL_) and charge transfer resistance (R_CT_) at the interface of electrolyte and active materials^36^, and diffusion of lithium ions within the solid phase of the electrode (R_SD_ + CPE_SD_). The first and second depressed semicircles (inset of Fig. 6b) with characteristic frequencies at ca. 9470 Hz and 455 Hz can be attributed to the SEI layers and charge transfer resistance, respectively^37^. The low frequency impedance tail can be ascribed to lithium diffusion in the electrolyte and active materials^35^, which is represented by CPE_LD_ and CPE_SD_ + R_SD_, respectively.

The results obtained from impedance data fitting demonstrate the stability of the NiO NWF anode. Charge-transfer resistance decreases by 38% between the first and the fifth cycle, which corresponds to the facilitation of lithium ion diffusion via electrolyte wetting. Resistance corresponding to the SEI layers (R_SEI_) stays constant throughout the initial cycles. This reveals the formation of stable passivating layers in the first cycle, which alleviates capacity loss with cycling. The ideality n of SEI layers decreases slightly from 0.85 to 0.78, denoting minimal structural change during the first few cycles. The idealities of double layer capacitance and the diffusion capacitances do not change much, and ESR keeps constant during the initial cycles, which also suggest improved stability of the electrode.

In conclusion, we have developed NiO-decorated Ni nanowire foam with solution-based synthesis, low temperature hydrogen reduction followed by air annealing process. NiO NWF has been shown as a perspective anode for Li-ion batteries. Excellent stability with minimal capacity fading over 1000 cycles with 680 mAh g^−1^ at 0.5C, and good rate capability at very high current rates (20C and 50C, with ca. 164 and 75 mAh g^−1^, respectively) indicate the superior electrochemical performance of the anode. Superb rate capability and stability can be evidenced with EIS results demonstrating low ESR of ca. 3.5 Ω and stable electrochemical parameters with cycling, respectively. Simple production procedures utilizing liquid-based solution, eco-benign compounds and low temperature render the mass manufacturing of the NiO NWF anode plausible.

## Additional Information

**How to cite this article**: Liu, C. *et al*. Template Free and Binderless NiO Nanowire Foam for Li-ion Battery Anodes with Long Cycle Life and Ultrahigh Rate Capability. *Sci. Rep.* **6**, 29183; doi: 10.1038/srep29183 (2016).

## Supplementary Material

Supplementary Information

## Figures and Tables

**Table 1:** EIS fitting parameters of NiO NWF anode after 1^st^, 3^rd^ and 5^th^ CV cycles.

**Cycle**	**R**_**s**_	**R**_**SEI**_	**R**_**CT**_	**CPE**_**SEI**_		**CPE**_**LD**_		**CPE**_**DL**_		**CPE**_**SD**_		**R**_**SD**_
	(Ω)	(Ω)	(Ω)	**Q (**μ**F s**^**n−1**^)	**n**	**Q (mF s**^**n−1**^)	**n**	**Q (**μ**F s**^**n−1**^)	**n**	**Q (mF s**^**n−1**^)	**n**	**(k**Ω)
1^st^	3.5	5	30	6	0.85	15	0.5	70	0.75	4.5	0.93	50
3^rd^	3.5	5	23	5	0.82	15	0.5	65	0.73	6	0.92	50
5^th^	3.5	5	18.5	7	0.78	16	0.5	60	0.75	8	0.92	50
