# Supplementary material for: Template Free and Binderless NiO Nanowire Foam for Li-ion Battery Anodes with Long Cycle Life and Ultrahigh Rate Capability
Source: Sci Rep. 2016 Jul 18;6:29183. doi: 10.1038/srep29183 (PMC4947918; doi:10.1038/srep29183)
Supplement: Supplementary Information [file srep29183-s1.doc]

**Supplementary Information**

**Template Free and Binderless NiO Nanowire Foam for Li-Ion Battery Anodes with Long Cycle Life and Ultrahigh Rate Capability**

**Chueh Liu,^a^ Changling Li,^a^ Kazi Ahmed,^b^ Zafer Mutlu,^a^ Cengiz S. Ozkan*^a,c^ and Mihrimah Ozkan*^a,b^**

^a^Materials Science and Engineering Program, University of California, Riverside, CA, USA

^b^Department of Electrical Engineering, University of California, Riverside, CA, USA

^c^Department of Mechanical Engineering, University of California, Riverside, CA, USA

Correspondence and requests for materials should be addressed to

C. S. O. (cozkan@engr.ucr.edu) and M. O. (mihri@ece.ucr.edu)


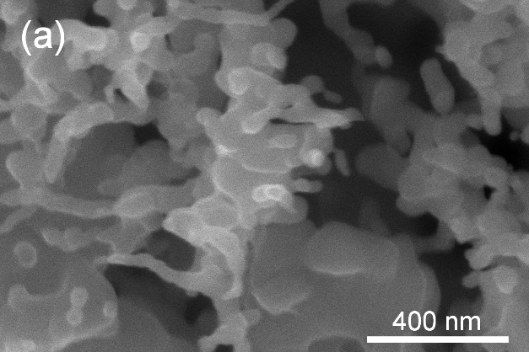

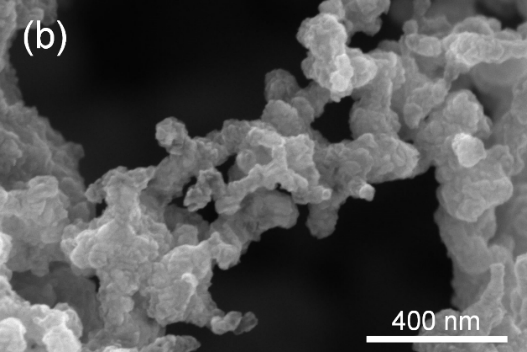

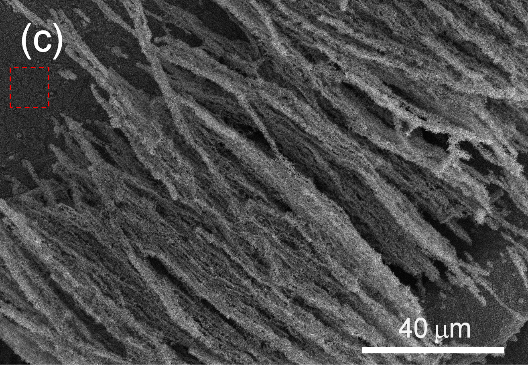

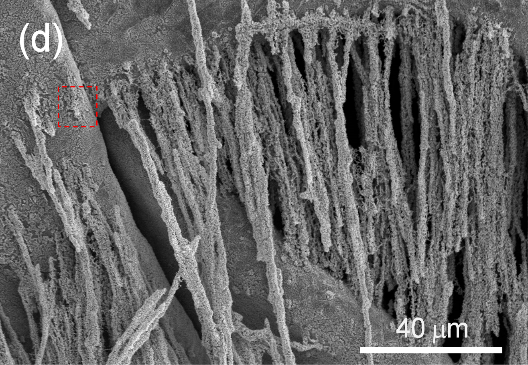

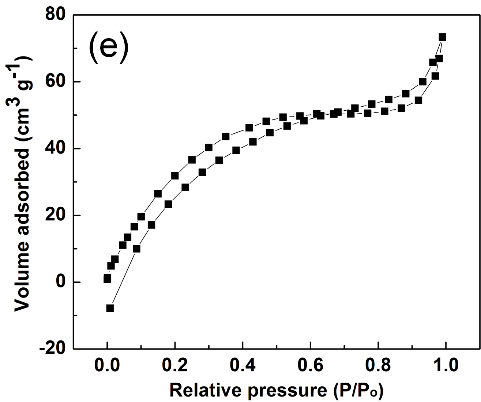

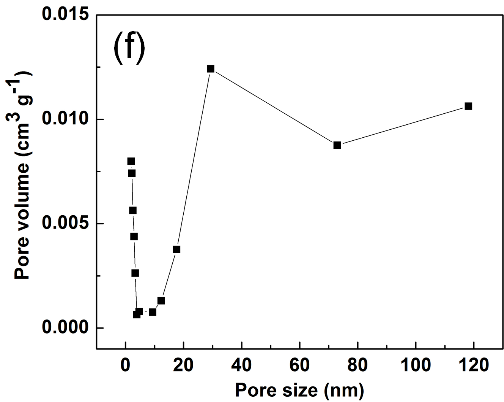


**Fig. S1** SEM images with large magnification for (a) Ni nanowire and (b) NiO nanowire foam, and low magnification for (c) Ni nanowire and (d) NiO nanowire foam, where the visual cues indicate Ni foam strut surface is coated with Ni and NiO nanowires, respectively. (e) Nitrogen adsorption–desorption isotherms and (f) pore size distribution of NiO nanowire foam.


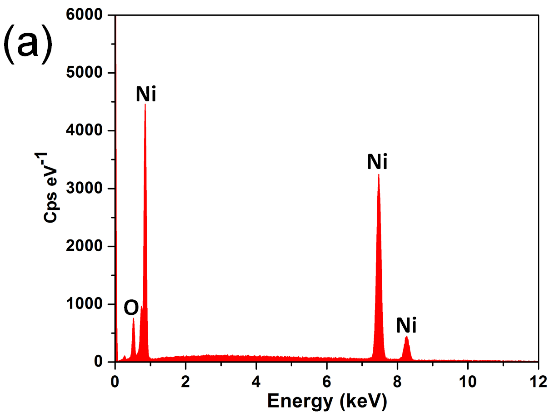

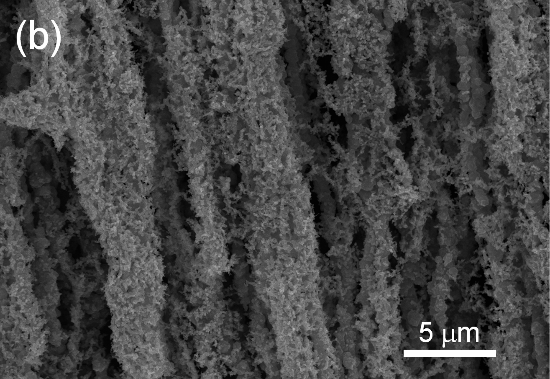

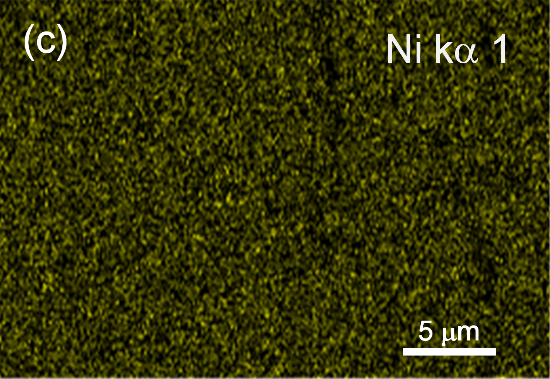

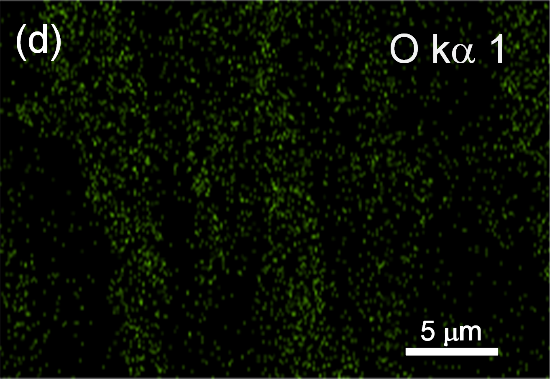


**Fig. S2** (a) EDX spectrum of NiO NWF. (b) Electron image of NiO NWF, and EDX elemental maps of (c) Ni and (d) O.


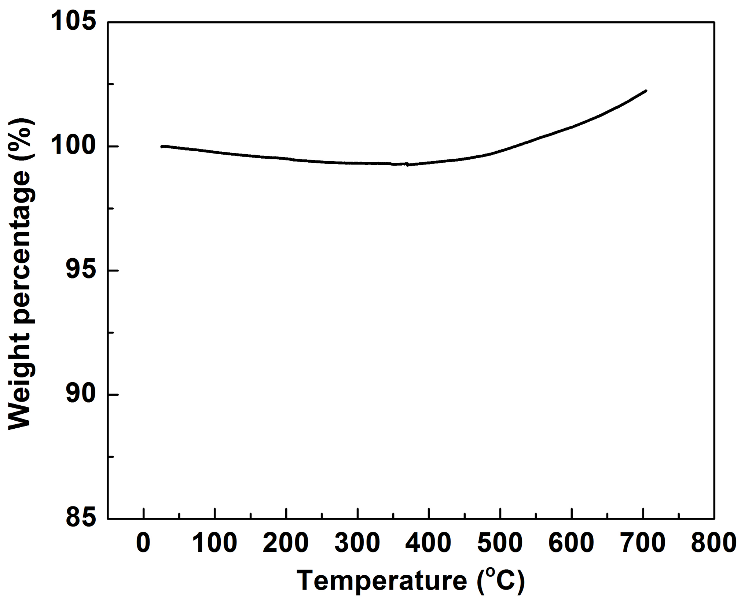


**Fig. S3** TGA curve of oxidation of Ni NWF into NiO NWF with air from room temperature to 700^o^C with 2^o^C min^-1^.


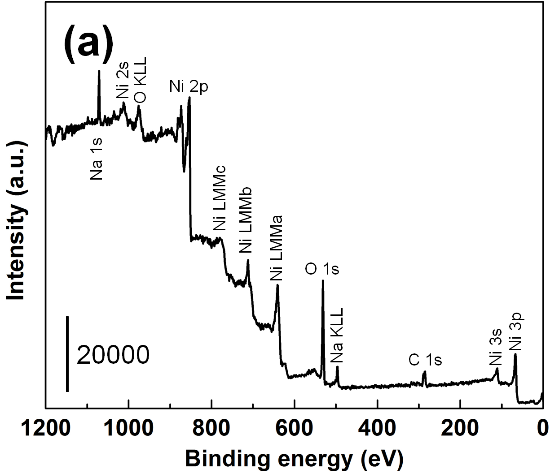

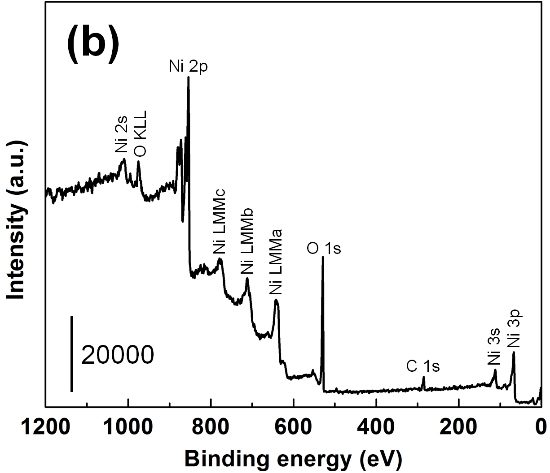


**Fig. S4** Survey XPS spectrum of (a) Ni NWF and (b) NiO NWF.


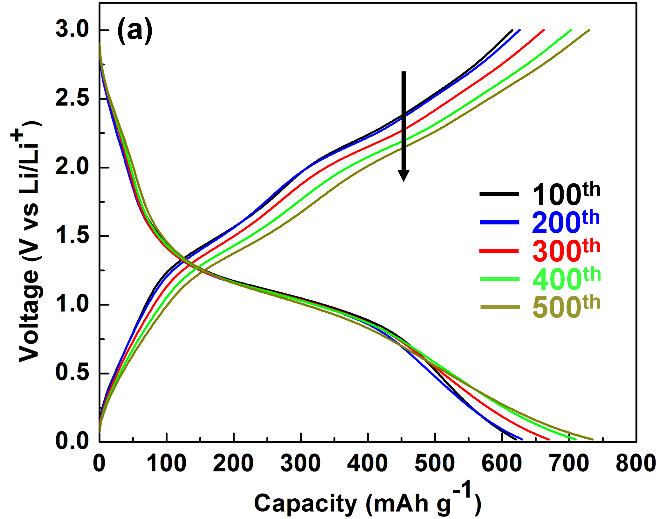

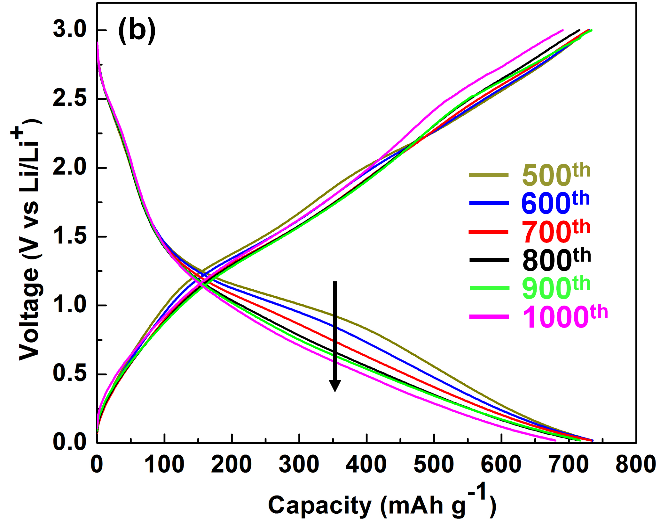


**Fig. S5** Charge-discharge curves of NiO NWF anode for (a) 100^th^ to 500^th^ cycles and (b) 500^th^ to 1000^th^ cycles.


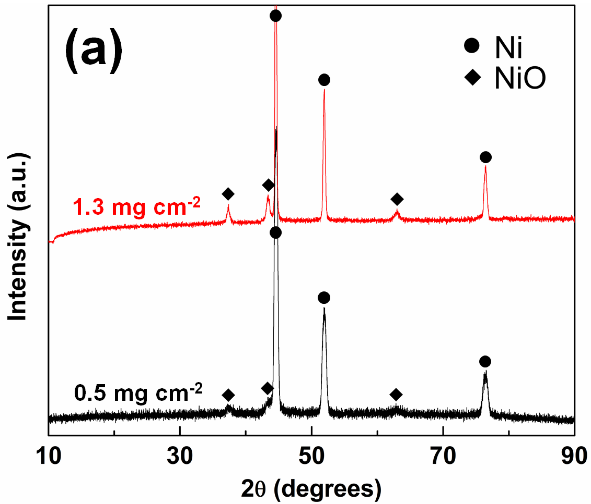

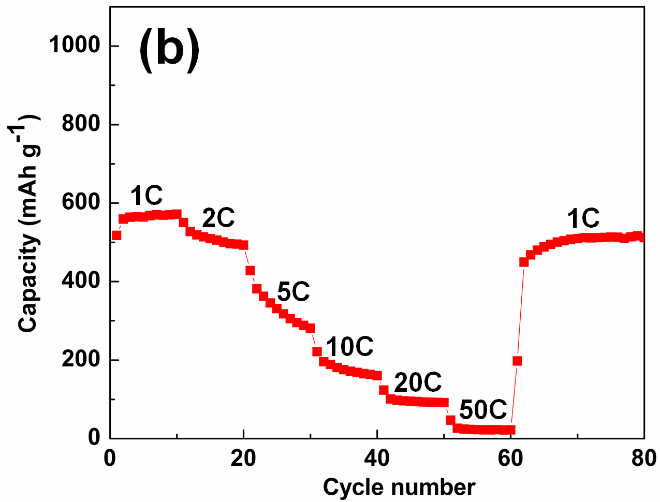


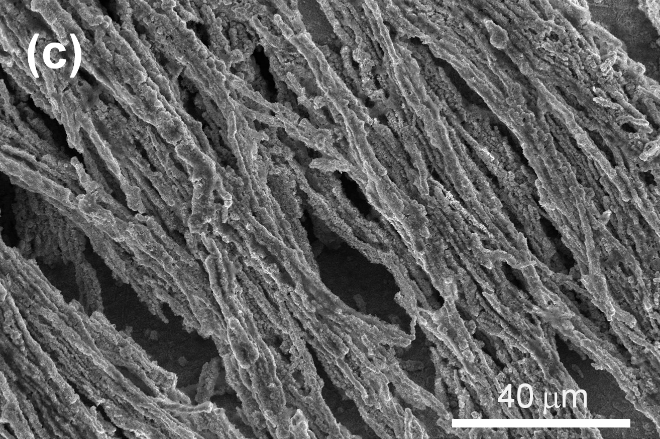

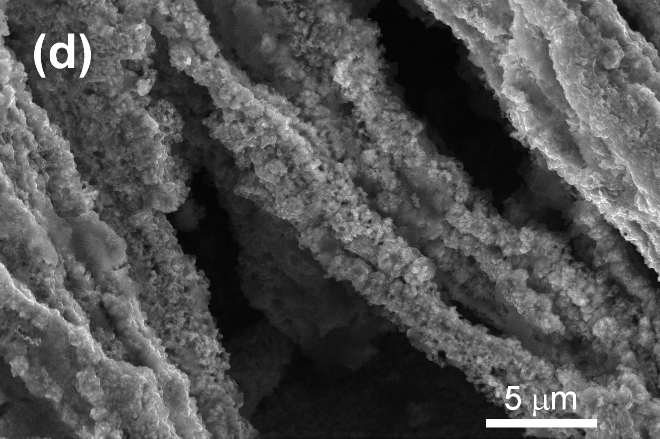

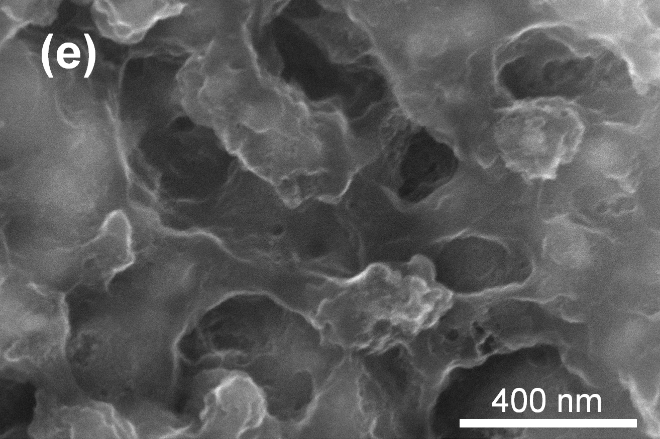


**Fig. S6** (a) XRD patterns of NiO NWF with 0.5 and 1.3 mg cm^-2^. (b) Discharge capacities of 1.3 mg cm^-2^ NiO NWF electrode at various current densities. (c-e) SEM images of NiO NWF after 1000 cycles.
